# Two-Way PBM–Euler Model for Gas and Liquid Flow in the Ladle

**DOI:** 10.3390/ma16103782

**Published:** 2023-05-17

**Authors:** Han Zhang, Hong Lei, Changyou Ding, Shifu Chen, Yuanyou Xiao, Qiang Li

**Affiliations:** 1 Key Laboratory of Electromagnetic Processing of Materials, Ministry of Education, Northeastern University, Shenyang 110004, China; 2010575@stu.neu.edu.cn (H.Z.);; 2 School of Metallurgy, Northeastern University, Shenyang 110004, China; 3 School of Chemistry and Chemical Engineering, Suzhou University, Suzhou 234000, China; 4 School of Materials and Metallurgy, University of Science and Technology Liaoning, Anshan 114000, China

**Keywords:** PBM, coalescence model, two-phase flow, bubble behavior, gas-stirred ladle, OpenFOAM

## Abstract

Ladle metallurgy is an important steelmaking technology in high-quality steel production. The blowing of argon at the ladle bottom has been applied in ladle metallurgy for several decades. Until now, the issue of breakage and coalescence among bubbles was still far from being solved. In order to have a deep insight into the complex process of fluid flow in the gas-stirred ladle, the Euler–Euler model and population balance model (PBM) are coupled to investigate the complex fluid flow in the gas-stirred ladle. Here, the Euler–Euler model is applied to predict the two-phase flow, and PBM is applied to predict the bubble and size distribution. The coalescence model, which considers turbulent eddy and bubble wake entrainment, is taken into account to determine the evolution of the bubble size. The numerical results show that if the mathematical model ignores the breakage of bubbles, the mathematical model gives the wrong bubble distribution. For bubble coalescence in the ladle, turbulent eddy coalescence is the main mode, and wake entrainment coalescence is the minor mode. Additionally, the number of the bubble-size group is a key parameter for describing the bubble behavior. The size group number 10 is recommended to predict the bubble-size distribution.

## 1. Introduction

Because of the need to meet the demand for high-quality steel, ladle metallurgy has received attention from many researchers [1,2]. Ladle refining is a popular steelmaking technology that has the following features: degassing, desulphurization, adjustment of the chemical composition of alloy elements, inclusion removal, as well as temperature homogenization [1,2,3,4]. Among ladle refining reactors, blowing argon at the ladle bottom is a widely used technology [4]. Due to the buoyancy, the bubbles rise upward and induce the recirculation flow to provide effective mixing and then escape from the free surface [2,3].

With the rapid development of computer hardware and software, some researchers devoted themselves to computational fluid dynamics (CFD). Currently, the relevant mathematical models include the quasi-single-phase model [5], the Euler–Lagrange model, and the Euler–Euler model [5,6,7]. The computational load of the quasi-single-phase model is the lowest among them, but the slip velocity between the liquid phase and the gas phase must be specified. The precondition of the Euler–Lagrange model is that the gas volume fraction is less than 12%. Additionally, the fluid flow is at a steady state, but the solution of the bubble trajectory equation is the function of the time, so the governing equations of the fluid phase have to be the unsteady differential equations. The Euler–Euler model takes into account the interaction between the gas phase and the liquid phase, and one set of partial differential equation equations corresponds to each phase, so the related computational load is heavy.

In many references, the bubble diameter is assumed to be constant. Certainly, such an assumption can simplify the multiphase mathematical model effectively [5,6,7]. But these models can not consider the breakup and coalescence of the bubble [3,4,8,9]. In fact, the complex motion of the bubbles affects the fluid flow significantly, and it is difficult to predict the spatial distributions of the gas fraction and gas velocity because these parameters are determined by the turbulent dispersion force, the drag force, the lift force, and the virtual mass force, and all these forces depend on the bubble diameter [10,11,12,13].

Therefore, some researchers began to apply the population balance model (PBM) to predict the size distribution of dispersed phases [14]. The combination of PBM and CFD can predict the breakup and aggregation of the inclusion, but such a model is rarely used to simulate bubble behavior in the gas-stirred ladle [15,16,17,18,19]. Morales et al. [20] used the volume of fraction (VOF) and PBM models to describe the multiphase flow, which is validated by the water-oil experiment. Li et al. [3] coupled the Eulerian multiphase flow model with the PBM to study the gas-liquid-slag three-phase flow in a water model ladle.

Although many researchers conducted interesting work regarding the transfer phenomena in the gas-stirred ladle, the issue of the effect of bubble behavior on the two-phase flow is still far from being solved. Firstly, these models have a heavy computational load due to the coupling of the three-phase Eulerian model and PBM. In this way, it is necessary to simplify the physical model. References [5,15] indicated that the mathematical model can ignore the effect of the top slag phase on fluid flow in the ladle. Secondly, the free surface in the ladle is always assumed to be flat. But in fact, the escape of gas bubbles from the free surface can lead to severe fluctuation of the free surface in the ladle. Thirdly, with the Eulerian model, the case of the bubble with a constant size was solved to obtain the fluid flow in the ladle. Then PBM is solved on the base of the known flow field to obtain the bubble-size distribution [9]. Such a one-way coupling results in the fact that the fluid flow is independent of the bubble breakage and bubble coalescence, which disagree with the fluid flow in the gas-stirred ladle. Fourthly, the turbulent eddy mechanism is assumed to be the only cause of the bubble coalescence. Such an assumption disagrees with the complex process in the gas-stirred ladle.

Inclusion behavior is another interesting issue in ladle metallurgy. The fluid flow and the bubble behavior affect the inclusion behaviors, which consist of inclusion transport with fluid flow, inclusion growth due to the collision among inclusions, and inclusion removal due to the flotation and attachment of the bubble. Some inclusion behaviors have been explained using various mathematical models. However, it is difficult to describe the inclusion behaviors in the ladle because of the rough flow field and the rough bubble behaviors [15,16,17,18,19].

The objective of this work is to obtain the transfer behavior of bubbles in the gas-stirred ladle using the PBM model and to predict the spatial distribution of bubble size and the gas volume fraction more reasonably. Therefore, in order to have a deep insight into the two-phase flow in the gas-stirred ladle, a two-way coupling mathematical model was developed to describe the interaction between the gas and the liquid in the ladle. This mathematical model consists of the Eulerian model and PBM. On the one hand, PBM is applied to describe bubble breakage and bubble coalescence, and bubble coalescence is involved in the turbulent eddy coalescence and wake entrainment coalescence. On the other hand, the Eulerian model is applied to describe the interaction between the bubble and fluid and to describe the behavior of the free surface.

## 2. Mathematical Model

### 2.1. Assumptions

The Euler–Euler model and PBM are used to describe the two-phase flow in the ladle with bottom blowing. In order to simplify the complex transfer process in the ladle, some assumptions must be applied in the mathematical model. (1) Both the gas and the liquid are the incompressible Newtonian fluid [1,2,3,4,5,6,7,8]; (2) The temperature of the molten steel stays constant [5]; (3) The effect of the top slag on the fluid flow is so weak that it can be ignored [5,6,7]; (4) The bubble is spherical [5,6]; (5) There are no chemical reactions in the molten steel [7,9]; (6) There is only binary breakage of the bubble [3,8].

### 2.2. Governing Equations

#### 2.2.1. Eulerian Multiphase Hydrodynamic Equations

The Euler–Euler model has a separate set of continuity and momentum equations for the gas phase and liquid phase, respectively [5,6,7,8,9]. The coupling of the phases comes from the shared interfacial momentum exchange source terms and the pressure field. The related physical parameters in the numerical simulation are listed in Table 1. The continuity equation and the momentum conservation model can be expressed as follows:

(1) $$\frac{\partial (\alpha_{k}\rho_{k})}{\partial t}+\nabla \cdot (\alpha_{k}\rho_{k}{\vec{u}}_{k})=0$$

(2) $$\frac{\partial (\alpha_{k}\rho_{k}{\vec{u}}_{k})}{\partial t}+\nabla \cdot \;(\alpha_{k}\rho_{k}{\vec{u}}_{k}{\vec{u}}_{k})=-\alpha_{k}\nabla P+\nabla \cdot \left[\alpha_{k}\mu_{\mathrm{eff},k}(\nabla {\vec{u}}_{k}+{\left(\nabla {\vec{u}}_{k}\right)}^{T})\right]+\alpha_{k}\rho_{k}\vec{g}+{\vec{R}}_{k}$$

where $\alpha_{k}$($\alpha_{g}+\alpha_{l}=1$), $\rho_{k}$ and ${\vec{u}}_{k}$ are the volume fraction, the density and velocity of gas (*k* = g), and liquid (*k* = l), respectively. *p* is the pressure, $\vec{g}$ is the gravity acceleration, and ${\vec{R}}_{k}$ is the interfacial momentum exchange source term between the gas and the liquid. $\mu_{\mathrm{eff},l}$ is the effective viscosity of liquid phase, which is determined with the standard k−ε turbulence model. The effective viscosity of gas phase, $\mu_{\mathrm{eff},g}$, is calculated using $\mu_{\mathrm{eff},g}=\frac{\rho_{g}}{\rho_{l}}\mu_{\mathrm{eff},l}$.

**Figure 1:**
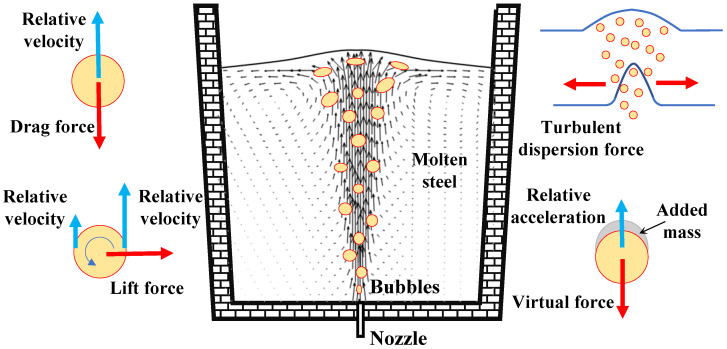
Schematic diagram of interphase forces in gas-stirred ladle.

The interfacial force source terms are expressed as:

(3) $${\vec{R}}_{l}=-{\vec{R}}_{g}={\vec{F}}_{D}+{\vec{F}}_{L}+{\vec{F}}_{\mathrm{VM}}+{\vec{F}}_{\mathrm{TD}}$$

where ${\vec{R}}_{l}$(${\vec{R}}_{g}$) denotes the interfacial momentum exchange source terms from the gas (liquid) phase to the liquid (gas) phase. ${\vec{F}}_{D}$, ${\vec{F}}_{L}$, ${\vec{F}}_{\mathrm{VM}}$, and ${\vec{F}}_{\mathrm{TD}}$ are the drag force, the lift force, the virtual mass force, and the turbulence dispersion force, respectively. Figure 1 shows the complex physical phenomena.

(1) The drag force, ${\vec{F}}_{D}$, which is the dominant interphase force, provides the resistance to the flow due to the motion of the bubbles relative to the molten steel, acts in the opposite direction to motion of the bubbles, and can be calculated with [21,22,23,24,25]:

(4) $${\vec{F}}_{D}=\frac{3}{4}\alpha_{g}\rho_{l}\frac{C_{D}}{d_{g}}\left|{\vec{u}}_{g}-{\vec{u}}_{l}\right|\left({\vec{u}}_{g}-{\vec{u}}_{l}\right)$$

where the Sauter mean diameter of bubbles $d_{g}$ is calculated from PBM. The drag coefficient $C_{D}$, which is given by Tomiyama [23],

(5) CD=max24Regmin1+0.15Reg0.687,3,8Eog3(Eog+4)

is related to the bubble Reynolds number (Reg=ρlu⇀g-u⇀ldg/μl), the bubble Eotvos number (${\mathrm{Eo}}_{g}=g\left|\rho_{l}-\rho_{g}\right|d_{g}{}^{2}/\sigma$), and the bubble Weber number (${\mathrm{We}}_{g}=\rho_{l}{\left|{\vec{u}}_{g}-{\vec{u}}_{l}\right|}^{2}d_{g}/\sigma$). $\mu_{l}$ is the viscosity of molten steel. σ is the surface tension coefficient. g is the magnitude of gravity.

(2) The interaction between the bubbles and the liquid shear field results in the lift force, ${\vec{F}}_{L}$, which is perpendicular to the relative motion of the bubbles and the liquid. The lift force is the function of the vector product of slip velocity and the curl of liquid velocity:

(6) $${\vec{F}}_{L}=-\alpha_{g}\rho_{l}C_{L}({\vec{u}}_{g}-{\vec{u}}_{l})\times (\nabla \times {\vec{u}}_{l})$$

where the lift coefficient $C_{L}$ is set to 0.1 [6].

(3) According to the Drew model [24,26], the virtual mass force, ${\vec{F}}_{\mathrm{VM}}$, arises from the inertia of molten steel relative to the acceleration of bubbles, and is defined as follows:

(7) $${\vec{F}}_{\mathrm{VM}}=\alpha_{g}\rho_{l}C_{\mathrm{VM}}(\frac{d{\vec{u}}_{g}}{dt}-\frac{d{\vec{u}}_{l}}{dt})$$

where the virtual mass coefficient $C_{\mathrm{VM}}$ is set to 0.5., $d_{g}/dt$ and $d_{l}/dt$ operators denote the substantial derivatives in the gas phase and the molten steel.

(4) The turbulent dispersion force, ${\vec{F}}_{\mathrm{TD}}$, comes from turbulent fluctuations in the molten steel velocity due to bubble-eddy interactions, which is given by Burns equations [27],

(8) $${\vec{F}}_{\mathrm{TD}}=\frac{3}{4}C_{D}\left|{\vec{u}}_{g}-{\vec{u}}_{l}\right|\frac{\mu_{t,l}}{0.9d_{g}}\alpha_{g}(\frac{\nabla \alpha_{g}}{\alpha_{g}}-\frac{\nabla \alpha_{l}}{\alpha_{l}})$$

where $C_{D}$ is the drag force coefficient, and $\mu_{t,l}$ is the turbulent viscosity of liquid phase.

#### 2.2.2. Population Balance Model

In order to predict the bubble-size distribution in the gas-string ladle, the population balance model (PBM) is employed, and the classes method (CM) [26,28,29,30] is implemented for solving PBM in OpenFOAM. The bubble distribution is represented by bubble classes, and the coalescence rate and breakage rate are transformed into the birth rate and death rate in each class. PBE for the *i*th bubble class can be given as follows:

(9) $$\frac{\partial }{\partial t}(\rho_{g}n_{i})+\nabla \cdot (\rho_{g}{\vec{u}}_{g}n_{i})=\rho_{g}(B_{C,i}-D_{C,i}+B_{B,i}-D_{B,i})$$

where $B_{C,i}$ and $B_{B,i}$ are not only the birth rates caused by the breakage and coalescence of bubbles, but also the death rates caused by the breakage and coalescence of bubbles. Bubble number density, *n_i_*, is the number of bubbles in group *i* per unit volume.

PBE has the same form as the generic convection-diffusion equation for multiphase flow.

(10) $$\frac{\partial }{\partial t}(\alpha_{g}\rho_{g}f_{i})+\nabla \cdot (\alpha_{g}\rho_{g}{\vec{u}}_{g}f_{i})=\rho_{g}v_{i}(B_{C,i}-D_{C,i}+B_{B,i}-D_{B,i})$$

Here, the bubble number density and bubble volume fraction of the dispersed phase have the following relationship:

*n*_*i*_*v*_*i*_ = *α*_g_*f*_*i*_ (11)

where *f_i_* is the volume fraction of size group *i* in the total dispersed phase fraction, *f_i_* = *α_i_*/*α*_g._ They satisfy $\sum_{i}f_{i}=1$ and $\alpha_{g}=\sum_{i}\alpha_{i}$. *v_i_* is the volume of a bubble with the diameter *d_i_*.

The source terms on the coalescence and breakage of the bubble can be modeled as

(12) $$B_{C,i}=\frac{1}{2}\int_{0}^{v}a(v-v^{\prime },v^{\prime })n(v-v^{\prime })n(v^{\prime })dv^{\prime }$$

(13) $$D_{C,i}=n(v)\int_{0}^{\infty }a(v\;,v^{\prime })n(v^{\prime })dv^{\prime }$$

(14) $$B_{B,i}=\int_{0}^{\infty }m(v^{\prime })b(v^{\prime })p(v\;,v^{\prime })n(v^{\prime })dv^{\prime }$$

(15) $$D_{B,i}=b(v)n(v)$$

where $a(v-v^{\prime },v^{\prime })$ represents the coalescence rate between the bubble of size *v* and the bubble of size $v^{\prime }$, $b(v^{\prime })$ is the breakage rate of a bubble of size $v^{\prime }$, $m(v^{\prime })$ is the number of daughter bubbles generated from the breakage of a bubble of size $v^{\prime }$, and $p(v\;,v^{\prime })$ is the probability density function for a bubble of size *v*, generated by the breakage of a bubble of size $v^{\prime }$.

(1) Coalescence kernel functions

In the previous literature, many researchers only considered the turbulent eddy mechanism [8]. However, the coalescence of bubbles resulting from wake entrainment is significant for the formation of large bubbles. Thus, there are the turbulent eddy mechanism and the bubble wake entrainment in the current paper.

The coalescence rate *a*(*v_i_*, *v_j_*) between a bubble of size *v_i_* and a bubble of size *v_j_* is usually expressed as

(16) $$a(v_{i},v_{j})=\omega_{C}(v_{i},v_{j})P_{C}(v_{i},v_{j})$$

where $\omega_{C}(v_{i},v_{j})$ is the collision frequency, and $P_{C}(v_{i},v_{j})$ is the coalescence probability.

The bubble collisions frequency resulting from turbulent eddy is calculated with

(17) $$\omega_{C,t}(v_{j},v_{i})=\frac{\pi }{4}{\left(d_{i}+d_{j}\right)}^{2}{\bar{u}}_{ij}$$

where ${\bar{u}}_{ij}={\left({\bar{u}}_{i}^{2}+{\bar{u}}_{j}^{2}\right)}^{1/2}$ is the characteristic velocity of the collision between bubbles of size *d_i_* and *d_j_*,. If the bubble size *d_i_* is equal to the eddy size, the mean turbulent velocity ${\bar{u}}_{i}$ of the bubble is equal to the mean turbulent velocity ${\bar{u}}_{\lambda }=\sqrt{2}{\left(\varepsilon_{l}\lambda \right)}^{1/3}$ of the eddy.

The bubble coalescence efficiency model is based on a phenomenological analysis. The coalescence between two bubbles depends on the ratio of the contact time *τ_ij_* and the coalescence time *t_ij_* for the drainage of the liquid film between them to reach a critical rupture thickness [8,27,31]. The coalescence probability function of the bubble of size *v_i_* and the bubble of size *v_j_* is written as

(18) $$P_{C,t}(v_{j},v_{i})=\exp\left(-\frac{t_{ij}}{\tau_{ij}}\right)=\exp\left\{-\frac{{\left[0.75(1+\xi_{ij}^{2})(1+\xi_{ij}^{3})\right]}^{1/2}}{(\rho_{g}/\rho_{l}+C_{\mathrm{VM}}{)}^{1/2}{\left(1+\xi_{ij}\right)}^{3}}{\left(\frac{\rho_{l}d_{i}u_{ij}^{2}}{\sigma }\right)}^{1/2}\right\}$$

where $\xi_{ij}=d_{i}/d_{j}$.

The bubble collision frequency resulting from wake entrainment can be calculated with

(19) $$\omega_{C,w}(v_{j},v_{i})=\alpha_{g}K\Theta d_{i}^{2}{\bar{u}}_{i,w}$$

where the parameter K is set to be 6.0., ${\bar{u}}_{i,W}=0.71{\left(gd_{i}\right)}^{1/2}$ is the rising velocity of the leading bubble. The big bubbles have the effective wake region for the bubble to coalesce, so the parameter Θ is valid for the bubbles larger than *d_c_* and can be expressed as $\Theta ={\left(d_{j}-d_{c}/2\right)}^{6}/[{\left(d_{j}-d_{c}/2\right)}^{6}+{\left(d_{c}/2\right)}^{6}],\;d_{j}\geq d_{c}/2,\;\mathrm{else}\;0.$ The bubble coalescence efficiency is given by Hibiki and Ishii [32],

(20) $$P_{C,w}(v_{j},v_{i})=\exp\left[-0.46{\left(\frac{\rho_{l}\varepsilon_{l}^{2/3}}{\sigma }\right)}^{1/2}{\left(\frac{d_{i}d_{j}}{d_{i}+d_{j}}\right)}^{5/6}\right]$$

(2) Breakup kernel functions

In the turbulent flow, the bubble breakage rate is determined by the interaction among bubbles with a size close to the turbulent eddy. Therefore, the turbulent eddy, whose size is much smaller than the bubble size, does not have enough energy to break the bubble. On the other hand, the turbulent eddy whose size is greater than the bubble size can merely carry bubbles and does not cause bubble breakage. The breakage rate is described as a product of collision frequency and breakage efficiency.

**Table 1:** Physical parameters of water models and industrial ladle.

Parameters	Sheng’s Water Model [33]	Anagbo’s Water Model [34]	Industrial Ladle
Diameter of model, m	0.5	0.5	2.6
Bath depth, m	0.42	0.4	2.8
Inner diameter, m	0.004	0.06	0.08
Inlet flow rate of gas, mL/s	50, 150	600	9000
Density of liquid, kg/m^3^	1000	1000	7800
Viscosity of liquid, Pa s	0.001	0.001	6.2 × 10^−3^
Density of gas, kg/m^3^	1.225	1.138	1.783
Viscosity of gas, Pa s	1.74 × 10^−5^	1.663 × 10^−5^	2.39 × 10^−5^
Surface tension, N/m	0.073	0.072	1.7

In the current paper, the breakup model is derived from theories of isotropic turbulence [26,31]. The breakup rate function proposed by Lehr [35] can be applied to describe the bubble breakup. In this case, a bubble of volume *v_j_* breaks into two bubbles, one of volume *v_i_* and the other of volume *v_j_*_−_ *v_i_*.

(21) $$\Omega_{B}(v_{j},v_{i})=0.5d_{j}^{*}{}^{5/3}\exp(-\frac{\sqrt{2}}{d_{j}^{*}{}^{3}})\frac{6}{\pi^{3/2}d_{i}^{*}{}^{3}}\exp(-\frac{9}{4}{[\ln(2^{2/5}d_{i}^{*})]}^{2})(1+\mathrm{erf}[\frac{3}{2}\ln(2^{1/15}d_{j}^{*}{)])}^{-1}\frac{1}{L^{3}T}$$

where $T={\left(\frac{\sigma }{\rho_{l}}\right)}^{2/5}\frac{1}{\varepsilon_{l}^{3/5}}$ is the time scale. $d^{*}=\frac{d}{L}$ is the dimensionless diameter, and $L={\left(\frac{\sigma }{\rho_{l}}\right)}^{3/5}\frac{1}{\varepsilon_{l}^{2/5}}$ is the length scale.

According to the definition of breakage kernel, $b(v^{\prime })$ and $p(v,v^{\prime })$ can be obtained with Luo’s model, $b(v^{\prime })=\frac{\int_{0}^{v^{\prime }}\Omega_{B}(v^{\prime },v)dv}{m(v^{\prime })}$, $p(v,v^{\prime })=\frac{\Omega_{B}(v^{\prime },v)}{\int_{0}^{v^{\prime }}\Omega_{B}(v^{\prime },v)dv}$.

According to the bubble sizes and volume fractions for various bubble classes, the Sauter mean diameter is defined as [36]

(22) $$d_{s}=\frac{1}{\sum_{i=1}^{n}(f_{i}/d_{i})}$$

### 2.3. Numerical Details

**Figure 2:**
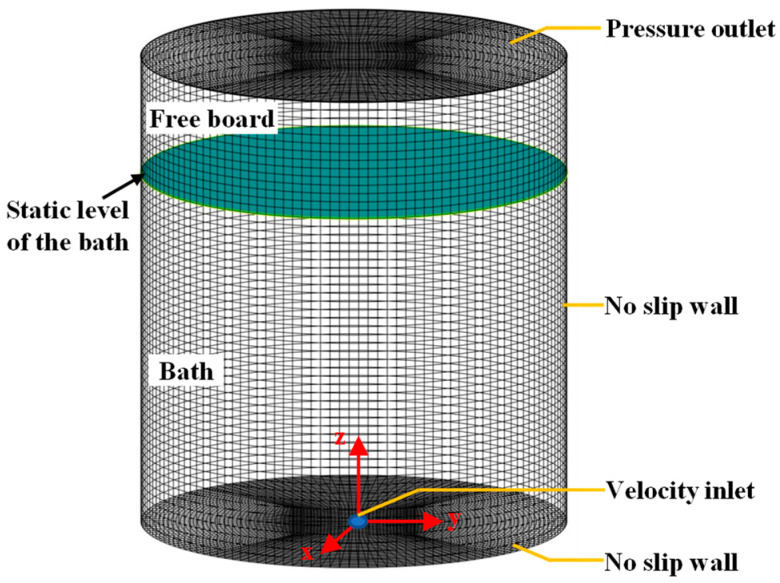
Grid system in gas-stirred ladle.

The numerical simulation for the gas-stirred ladle is carried out using the free, open-source software OpenFOAM (version 6.0.0). The computational domain includes the two-phase flow region and free board above the liquid in the ladle. The free-board height is one-third of the bath height. All the computational domains are covered with non-uniform hexahedral grids, as shown in Figure 2. The convergence criterion is that the root mean-square normalized residual is less than 10^−5^. The time step is set to 0.001 s.

There are five types of boundary conditions. The velocity inlet is determined by the total gas flow rate at the nozzle of ladle bottom, the pressure outlet is specified, the no-slip boundary condition is applied to the solid wall, and the pressure outlet is used for the gas phase and liquid phase, as shown in Figure 2. The detailed information is listed in Table 1.

Sano and Mori’s empirical equation is used to calculate the initial bubble size at the nozzle exit,

(23) $$d_{b}={\left[{\left(\frac{6\sigma d_{n}}{\rho_{l}g}\right)}^{2}+0.0242{\left(V_{g}^{2}d_{n}\right)}^{0.867}\right]}^{1/6}$$

where *d*_n_ is the nozzle diameter, and *V*_g_ is the gas flowrate.

## 3. Model Verification

### 3.1. Validation of Grid Independence

**Figure 3:**
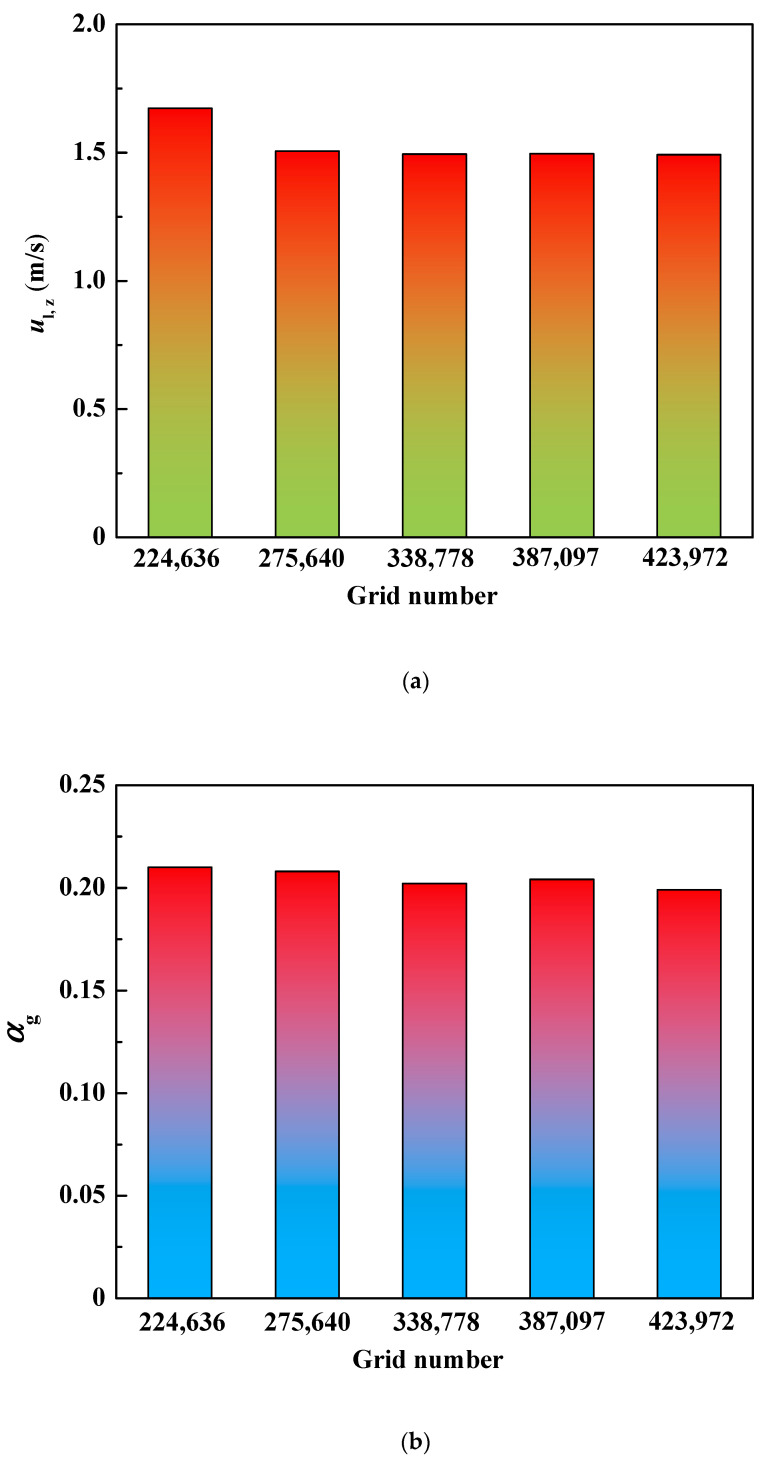
Verification of grid independence. (**a**) Liquid axial velocity, (**b**) gas volume fraction.

In order to ensure that the numerical results are not the spurious artifacts of poorly resolved grids, we carried out the grid sensitivity experiments for the industrial ladle with an inlet flow rate of 9000 mL/s. Based on the five grid systems, Figure 3 gives the maximum velocity and maximum gas volume fraction of molten steel at 1 m below the free surface.

The variation of the velocity and the variation of the gas volume fraction is 0.179 m/s and 0.002 between the grid numbers 224,636 and 275,640. When the grid is refined from 275,640 to 423,972, the variation of the velocity between other adjacent grids is 0.012 m/s, 0.002 m/s, and 0.006 m/s, and the gas volume fraction differences between other adjacent grids are 0.006, 0.002, and 0.005. There are no perceptible differences between the velocity and the gas volume fraction in the cases of 338,778 grids and 423,972 grids. Therefore, the 338,778 grids are fine enough for the coupling of the flow field and PBM.

### 3.2. Validation of Bubble-Size Distribution

The bubble-size distribution and the flow field reported in the literature [33,34] are applied to validate the numerical result. Table 1 gives the related geometrical parameters and physical parameters. 

In Anagbo’s experiment [34], the water model was a one-sixth scale model of a 150-ton steelmaking ladle. The experimental apparatus consisted of a cylindrical plexiglass tank containing deionized water with a depth of 0.4 mm and a porous plug located at the center of the bottom. The inlet flow rate was 600 mL/s. Additionally, the axial bubble diameter was measured using the double-contact electro-resistivity probe.

**Figure 4:**
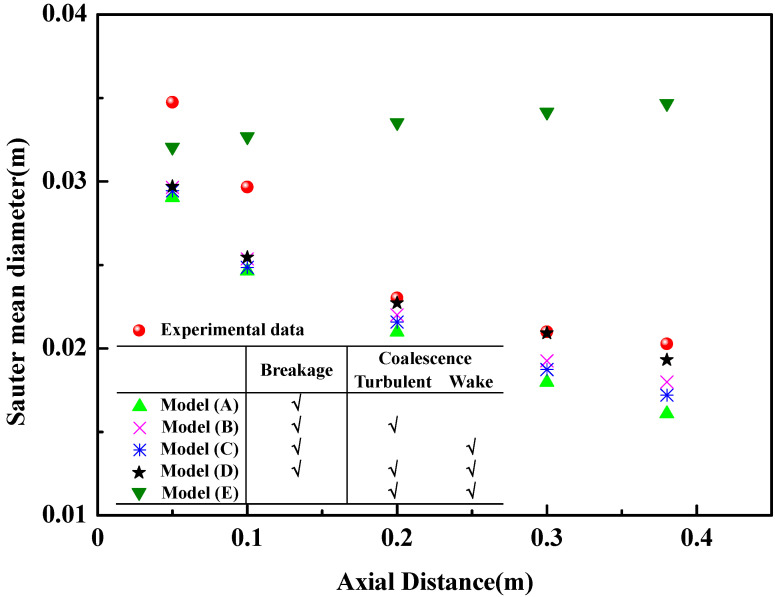
Validation of bubble size.

Figure 4 gives the difference between the experimental data and the numerical results: (1) The bubble behaviors consist of the bubble breakage and the coalescence among bubbles, and the coalescence among bubbles has two modes: turbulent eddy coalescence and wake entrainment. Model E only considers the coalescence and ignores the breakage, so the bubble diameter predicted with model E is the greatest among the models, and the bubble diameter increases with the increase in the axial distance. Such a phenomenon disagrees with the experimental data. (2) Model A only considers the breakage and does not consider the coalescence, so the bubble diameter predicted with model A is the least in all of the models. (3) If the mathematical model considers the breakage (Models A–D), the related numerical results are quite similar, and the predicted bubble diameters decrease with the increase in the axial distance. Such a phenomenon follows the experimental data. In other words, bubble breakage is an important feature of fluid flow in the ladle. (4) Model B (or model C) only considers turbulent eddy coalescence (or wake entrainment coalescence). The bubble size predicted with model B is greater than that by model C, so the effect of the turbulent eddy on the coalescence among bubbles is greater than wake entrainment. (5) Model D considers turbulent eddy coalescence and wake entrainment coalescence, so the bubble size predicted with model D is greater than that by model B (or model C). The bubble maximum diameter predicted with model (D) is 0.03 m, and the related experimental data are 0.034 m. In this way, the relative error is 11.8%. Several reasons lead to such a difference: (1) There is a spherical bubble assumption in the mathematical model. (2) It is difficult for the probe to identify spherical bubbles and non-spherical bubbles.

### 3.3. Validation of Flow Field

**Figure 5:**
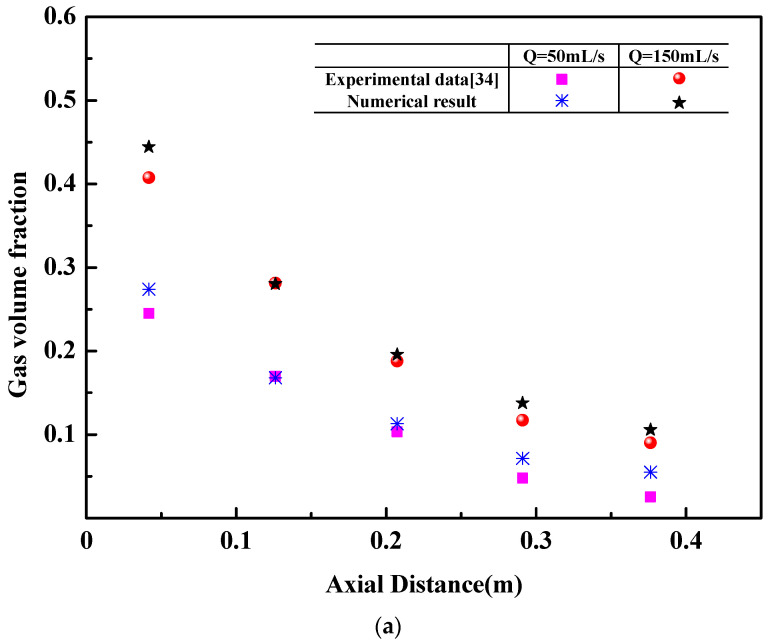

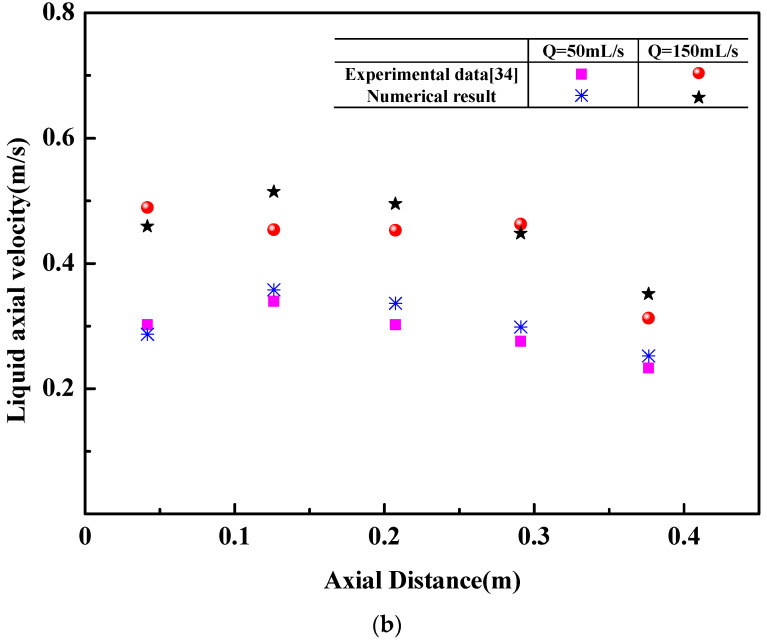
Validation of flow field. (**a**) Gas volume fraction, (**b**) liquid axial velocity.

Figure 5 shows that the numerical results agree well with experimental data [33]. In the water model of a geometrical scale of 1/10, a flush-mounted orifice with a 4 mm inner diameter is placed at the center of the ladle bottom, and the measurements are made using a combined electrical probe technique and the laser Doppler anemometer (LDA). Figure 5a indicates that the predicted maximum gas volume fraction is 0.44, the related experimental value is 0.41, and the relative error is 7%. Figure 5b indicates the maximum difference of liquid axial velocity between the experimental data and the predicted result is 0.04 m/s, and the maximum relative error is 12%.

## 4. Results and Discussion

### 4.1. Bubble-Size Distribution for Different Bubble-Size Classes

In order to balance the computational cost and the computational accuracy, the Sauter mean diameter of argon bubbles at the ladle axis is applied in the four bubble-size groups. Based on the Sauter mean diameter, the bubble size is divided into G groups, $v_{i+1}=\theta v_{i}\;(i=1,\;2,\;3,\;\cdots G-1)$. 

**Figure 6:**
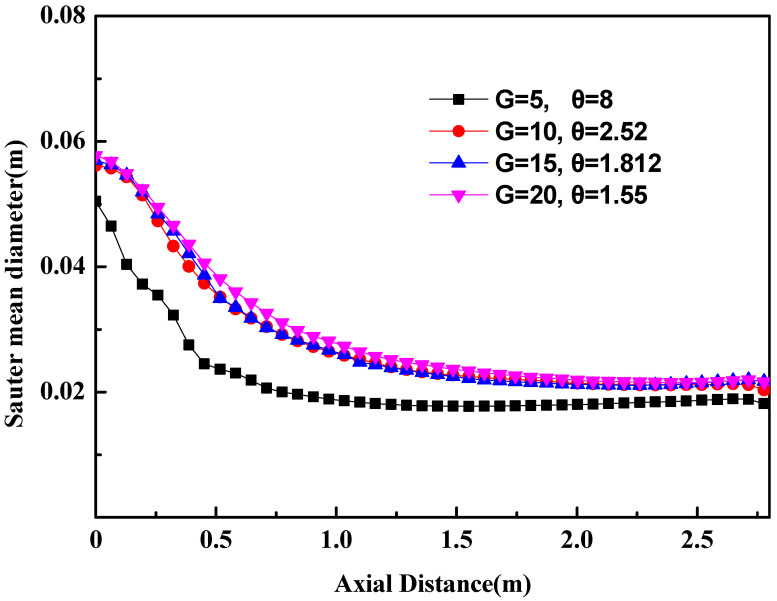
Bubble-size distribution.

The Sauter mean diameter of argon bubbles has the following features at the ladle axis, as shown in Figure 6: (1) The diameter of argon bubbles at the ladle axis follows a similar trend for the four bubble-size groups. The bubble diameter decreases in the initial rising stages and then remains unchanged gradually. Such a distribution feature was observed by Anagbo [34]. (2) In the four cases, the diameter of argon bubbles in the case of G = 5 is the smallest because the bigger geometric discretization factor θ leads to the greater error. (3) The distribution of the diameter of argon bubbles is almost similar in the cases of G = 10, 15, 20. The maximum relative error of the Sauter mean diameter of argon bubbles between M = 10 and M = 20 is only 8.8%. Thus, the bubble size is divided into ten groups in the following numerical calculation. In the previous paper [3,11], the geometric discretization factor on bubble size was not considered, and the bubble was directly divided into fixed groups in the numerical calculation. Such a simplification may lead to a bad result, such a simplification may lead to a bad result that the predicted bubble size is less than the actual bubble size.

### 4.2. Flow Field in the Industrial Ladle

**Figure 7:**
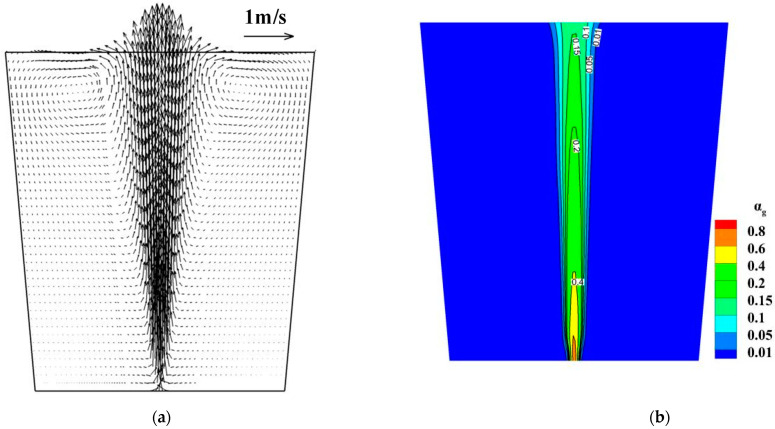
Two-phase flow in the ladle. (**a**) Velocity of molten steel, (**b**) gas volume fraction.

The flow field of molten steel and the argon volume fraction in the ladle are shown in Figure 7. Argon bubbles leave the ladle bottom as a strong jet flow and spread outward until they reach the free surface. As argon bubbles float upward, the gas volume fraction decreases gradually, but the plume region increases gradually. Because of the buoyancy of argon bubbles, the molten steel also flows upwards. Consequently, there are vortexes on both sides of the gas jet flow, and these vortexes are near the free surface. Some researchers [37,38] used the Euler–Euler approach to describe the fluid flow in the gas-stirred ladle and indicated that the width of the plume remained almost unchanged along the vertical direction. Such a result is different from the experimental result. The reason is that their mathematical model ignored the effect of the turbulent dissipation force on the fluid flow.

### 4.3. Disperse Phase in the Industrial Ladle

**Figure 8:**
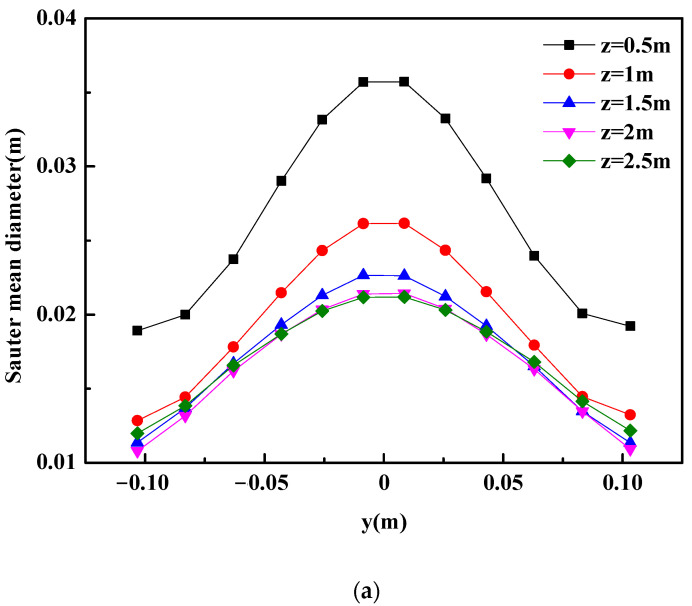

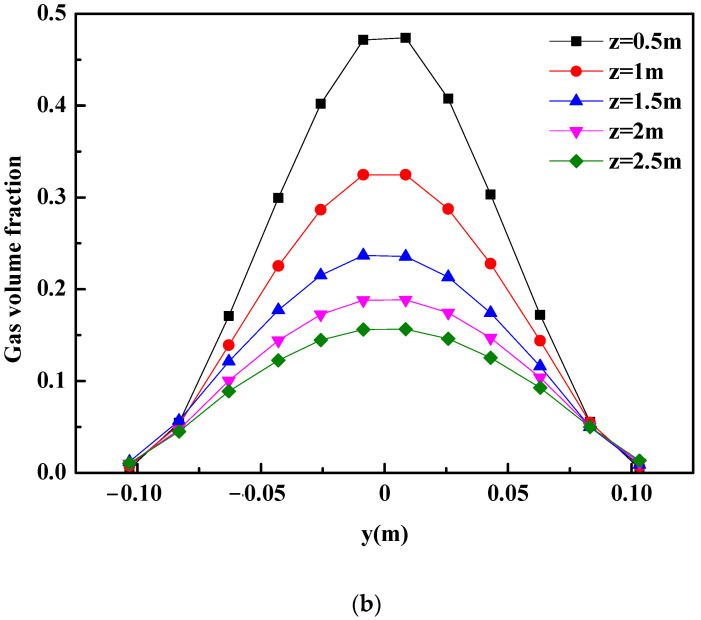
Gas behaviors in the ladle. (**a**) Bubble size and (**b**) gas volume fraction in the plume region.

It can be observed in Figure 8 that the bubble diameter and the gas volume fraction have some similar features in the plume region of the industrial ladle: (1) The bubble diameter and the gas volume fraction are symmetrically distributed on both sides of the ladle centerline. (2) The bubble diameter and the gas volume fraction along the plume centerline are greater than that in other regions. (3) The bubble diameter and the gas volume fraction gradually decrease with the increase in the axial distance from the ladle bottom. 

### 4.4. The Effect of Bubble Size on Flow Field in the Industrial Ladle

**Figure 9:**
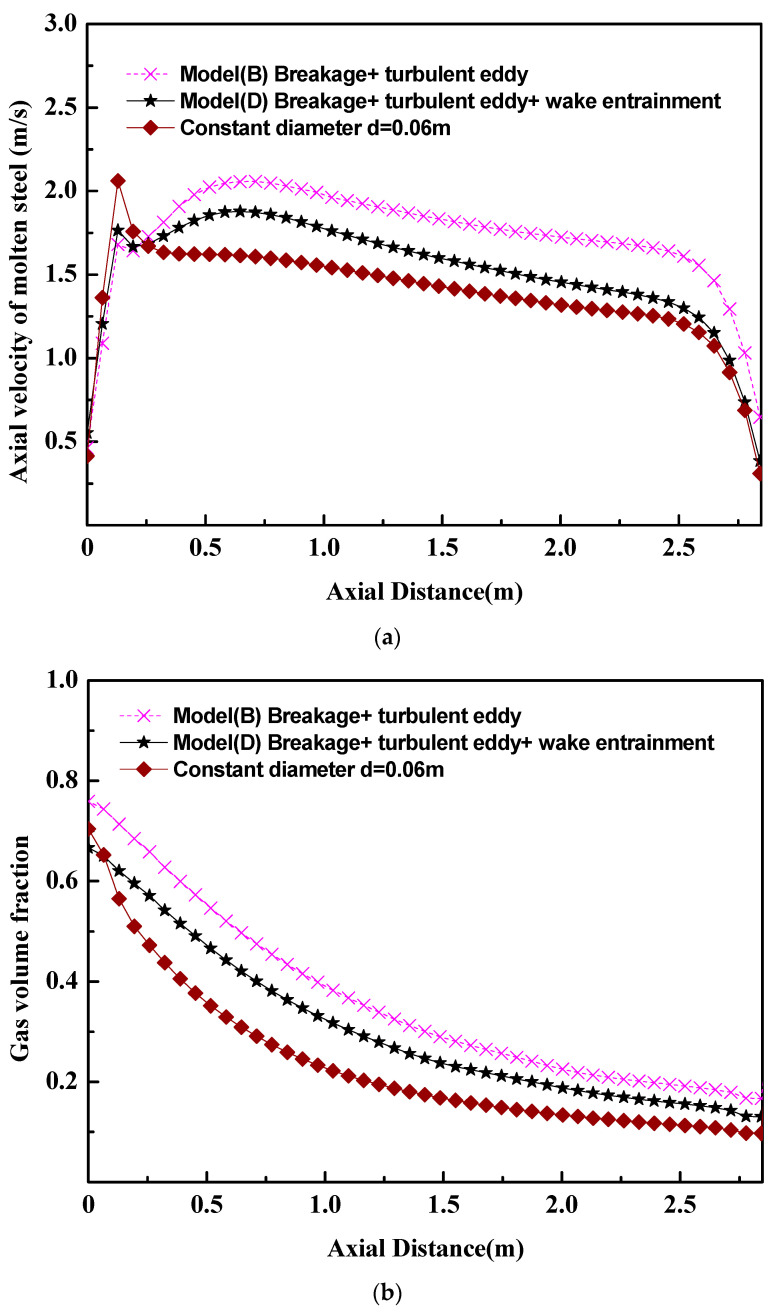

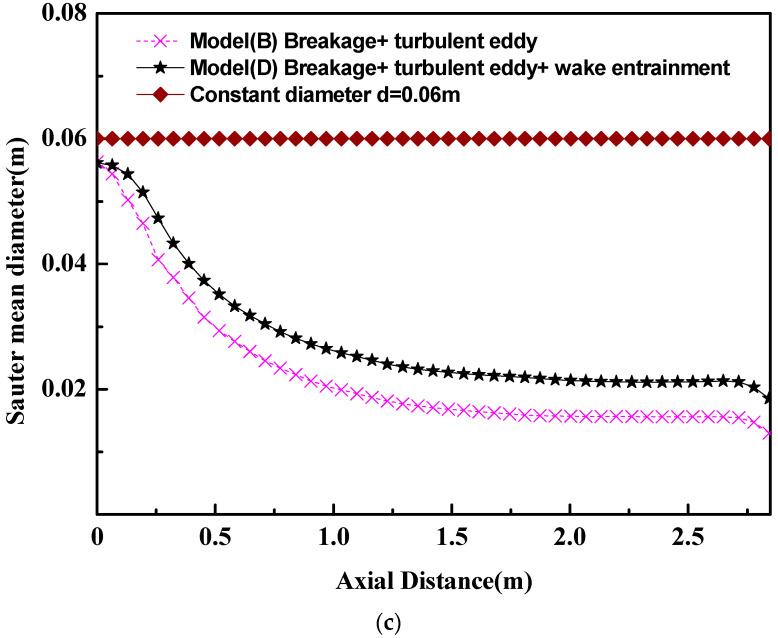
The effect of bubble’s breakage and coalescence on the two-phase flow in the ladle. (**a**) Axial velocity of molten steel, (**b**) gas volume fraction, (**c**) bubble size at the ladle axis.

In the past references [5,6,7], there were many research works based on the constant bubble size or the turbulent eddy coalescence among bubbles. Figure 9 gives the differences among the mathematical models with a constant bubble size, the mathematical model with a bubble turbulent eddy coalescence (model B), and the mathematical model with a bubble turbulent eddy coalescence and wake entrainment coalescence (model D). Figure 9a shows that, in the case of the constant bubble size, there is an abrupt increase in the axial velocity of molten steel. But an abrupt increase in the axial velocity of molten steel is not obvious in the cases of model B and model D. Such a difference comes from the effect of bubble breakage and bubble coalescence on the flow of molten steel. Figure 9b also shows that, with the increase in the distance from the ladle bottom, the gas volume fraction in the case of the constant bubble size decreases faster than that in the case of model B and model D. Such phenomena indicate that the interaction between the fluid flow and the bubble breakage/coalescence can prompt the uniformity of fluid velocity and bubble distribution. Figure 4 and Figure 9c show the importance of the wake entrainment coalescence of bubbles. In other words, the bubble behaviors can be described accurately only when the bubble breakage and the bubble coalescence (turbulent eddy and wake entrainment) are considered in the mathematical model.

## 5. Conclusions

In order to solve the issue of breakage and coalescence among the bubbles in ladle metallurgy, this work proposed a strategy that the Euler–Euler model and population balance model are coupled to investigate the complex fluid flow in the gas-stirred ladle. Research shows that there is coalescence and the breakage of bubbles in the molten steel in the ladle. Bubble breakage is a necessary factor. If the mathematical model ignores this factor, the mathematical model gives the wrong bubble distribution. Furthermore, the bubble coalescence in the ladle consists of turbulent eddy coalescence and wake entrainment coalescence. The turbulent eddy coalescence is the main mode, and the wake entrainment coalescence is the minor mode. The bubble breakage, turbulent eddy coalescence, and wake entrainment coalescence are introduced to describe bubble behavior in order to ensure that PBM can give the transfer behavior in the ladle accurately. Meanwhile, it is clarified that the number of the bubble-size group is the key parameter to describe the bubble behavior, and the size group number 10 is recommended to predict the bubble-size distribution in the ladle.

## Data Availability

No new data were created or analyzed in this study. Data sharing is not applicable to this article.
